# A compact detector system for simultaneous measurements of the light yield non-linearity and timing properties of scintillators

**DOI:** 10.1038/s41598-024-57186-9

**Published:** 2024-03-23

**Authors:** Benoit Sabot, Chavdar Dutsov, Philippe Cassette, Krasimir Mitev, Matthieu Hamel, Guillaume H. V. Bertrand, Kheirreddine Lebbou, Christophe Dujardin

**Affiliations:** 1 CEA, LIST, Laboratoire National Henri Becquerel (LNE-LNHB), Université Paris-Saclay, 91120 Palaiseau, France; 2 Faculty of Physics, Sofia University “St. Kliment Ohridski”, 1164 Sofia, Bulgaria; 3 CEA, LIST, Université Paris-Saclay, 91120 Palaiseau, France; 4 Institut Lumière Matière UMR 5306 CNRS, Universite Claude Bernard Lyon 1, 69622 Villeurbanne, France; 5 Institut Universitaire de France (IUF), 75231 Paris, France; 6 Present Address: Paul Scherrer Institute, 5232 Villigen, Switzerland

**Keywords:** Experimental nuclear physics, Fluorescence spectroscopy, Photonic devices, Characterization and analytical techniques

## Abstract

This work presents an outline of a detection system that employs the Compton spectrometer method to assess the non-linearity of scintillator light yield. A novel approach is introduced, leading to more accurate measurements through the separate determination of the intrinsic light output parameters and the non-linearity of the scintillators. Key features of this system include the use of a portable scintillation detector with three photomultiplier tubes for precise measurement of the average number of detected photoelectrons and the incorporation of recent advancements in correction techniques for accidental coincidences. The integration of digital acquisition, offline data analysis, and geometric adaptation reduces the time required to perform a measurement. The developed detector can simultaneously measure different timing properties, as well as the relative intensities following ionization excitation in a scintillator. The system’s performance is demonstrated through measurements of the light yield dependence on the deposited energy for commercially available liquid, plastic, and inorganic scintillators. Such instrumentation serves as a valuable tool in the development of novel scintillating materials, including liquid or solid organic scintillators, inorganic scintillators, and composite scintillators for electron detection, in addition to traditional X-ray or $\gamma$-ray detection.

## Introduction

Scintillation is used to convert ionising radiation into light that can be detected by light detectors, such as photomultiplier tubes (PMTs), which then further convert it into electrical signals. There are several types of scintillators, with the most common being organic liquid cocktails, plastic scintillators, or inorganic single crystal scintillators. All of them exhibit the same phenomenon: non-linearity in response depending on the energy and type of radiation interacting with the material. Currently, the characterisation of these scintillators is challenging, and they are typically compared with each other using relative measurements or techniques. This problem also applies to scintillating materials in different categories, such as liquid, organic, and inorganic. Another issue is the dependence of scintillator properties, such as light yield or pulse shape discrimination capability, on the scintillator’s morphology. For instance, Yttrium aluminum garnet activated by cerium (YAG:Ce) as a single crystal or nano-structured material (e.g., aerogel) may exhibit different behavior, particularly those developed for new means of detecting pure beta emitter radioactive gases^1^ (especially $^3$H). However, since this represents a new type of scintillating material, determining their scintillation characteristics is fundamentally different from characterizing, for example, an inorganic scintillator crystal like bulk YAG:Ce. Consequently, in the context of developing these new scintillators, there is a need to create instrumentation capable of characterizing the response of scintillators with respect to timing and scintillation yield. One of the priorities is studying low energies, such as those corresponding to electrons emitted by $^3$H. The objectives of this study were to design portable instrumentation capable of simultaneously measuring a scintillator’s response and its temporal properties. This work introduces an alternative approach to characterizing scintillator response, which differs from the conventional techniques used in previous studies^2–6^, as it employs a compact Compton-TDCR method. The Triple to Double Coincidence Ratio (TDCR)^7^ enables precise and absolute measurement of the number of photoelectrons. By employing the coincidence measurement of TDCR events and a gamma-ray detector to detect the Compton spectrum, we can precisely determine the scintillator response as a function of the energy of the electrons interacting in the medium and the zone of primary interaction for inorganic scintillators. This study presents the design of a device based on our recent works^8^, focusing specifically on geometric optimization and the implementation of instrumentation that enables offline data processing. These advancements, coupled with recent efforts in correcting accidental coincidences^9^, enable highly precise measurements to be obtained within a relatively short timeframe (less than 48 hours). The capability for offline processing also allows for measuring the scintillator’s temporal response within a selected energy range, which is the primary objective of this study. To demonstrate the device’s capabilities, we present results obtained from various commercial and laboratory-made liquid and plastic scintillators, as well as samples of inorganic scintillators. This application is not only highly relevant in the field of ionizing radiation metrology but also for conducting detailed investigations into scintillation mechanisms. It will significantly contribute to the in-depth study of scintillation models and allow testing of simple and complex quenching models or even more complex ones developed in the past or under study^10^.”

## Results

### Measurement of the scintillation yield of liquid scintillators

The Compton-TDCR system (whose design is described in Compton coincidences system design) was employed to measure three commonly used commercial Liquid Scintillation Cocktails (LSC): Ultima Gold™ (UG), Ultima Gold™Levels of Tritium (UG LLT), and Hionic-Fluor™(HF). Measurements were also conducted on a laboratory-prepared liquid scintillation cocktail composed mainly of Toluene + PPO, with the composition and characteristics, detailed in Sect. "Organic scintillator composition and preparation".

**Figure 1:**
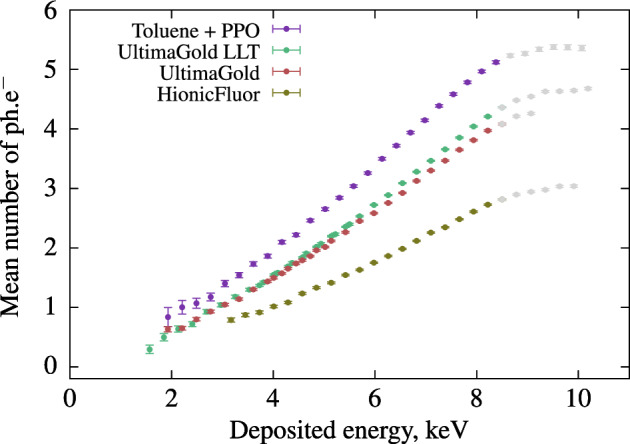
Relative light output of Ultima Gold, Ultima Gold LLT, toluene + PPO and HionicFluor cocktails. The data above 8.5 keV are not trusted as described in Sect. "Measurement data analysis for the scintillator non-proportionality".

The mean number of detected photo-electrons as a function of the deposited energy in the cocktails are shown in Fig. 1. The data analysis was carried out in accordance with the established method (Sect. "Measurement data analysis for the scintillator non-proportionalit") and from these raw data, we can deduce the physical limitations of the current configuration. Beyond a certain threshold of energy deposited in the scintillator, the Compton phenomenon becomes disturbed by counts related to double Compton events, leading to an inaccurate estimation of the number of photons produced as a function of deposited energy. Conversely, when the deposited energy is very low, close to the first Compton events, the number of photons is difficult to measure due to low statistics.Worse still, as we approach the $^{241}$Am peak, in the case of a few photoelectric events, the measurement becomes erroneous, limiting, in this configuration, the threshold just below 2 keV at best. Near the photoelectric peak of $^{241}$Am , the counting statistics amount to only a few counts per hour, and, on occasion, we even observe Am-241 photoelectric events measured on the detector coincident with photons. We believe that this is an artifact of false coincidences, which may be induced either by a poorly collimated part of the beam (not identified in our Monte Carlo simulations) or by Rayleigh scattering without energy loss in the scintillator. On the whole, the initial results indicate that the toluene-based cocktail has the highest light output among all the measured LSCs. The light output of the HF cocktail is considerably lower than that of the other samples. The curves for UG and UG LLT are obtained by combining two measurements, one at an angle of 40$^\circ$ and the other at 90$^\circ$ between the source and detector. This approach is used to broaden the range of energies studied, which also serves as evidence for a well-designed concept, as described in the method (Sect. "Measurement data analysis for the scintillator non-proportionalit"). For both samples, the light output is very similar between 2 and 4 keV, but non-proportionality becomes more significant for LLT than for UG as the deposited energy increases. Based on these initial measurements, the system demonstrates its capability to determine the relative light output of cocktails within the energy range of 2 keV to 7.5 keV (Energy range limits described in Sect. Measurement data analysis for the scintillator non-proportionalit). In this scenario, the scintillators emit precisely at the wavelength corresponding to the peak quantum efficiency of the photomultipliers, making it the optimum choice. Nevertheless, it becomes useful for studying the response of LS cocktails when measuring low-energy emitters such as $^3$H ($\beta ^-_{mean}$ = 5.68 keV), $^{55}$Fe (5 to 6 keV X-rays and Auger electrons), or $^{241}$Pu ($\beta ^-_{mean}$ = 5.10 keV). To achieve precise measurements in the higher energy range, it is possible to employ a gamma source with higher energy. However, in this study, the choice of gamma source was restricted to $^{241}$Am in order to validate the concept and to be closer to our targeted energy of the electrons of $^3$H.”

### Light output measurements with filters and approximation with Birks’ ionization quenching formula

Birks’ semi-empirical ionization quenching formula is the most widely used equation for describing the non-linearity of organic scintillators. This formula relates the light output of the scintillator, denoted as *L*, to the deposited energy *E*, and it is defined by Birks^11^.

1 $$\begin{aligned} L = \int _0^E \frac{dE'}{1 + kB(dE'/dx)}, \end{aligned}$$

where $dE'/dx$ is the stopping power of the electron for energy $E'$ and *kB* is Birks’ ionization quenching factor which is specific to the cocktail and is measured in units µmMeV$^{-1}$. The mean number of detected photo-electrons is then:

2 $$\begin{aligned} {\bar{n}}(E) = S~L, \end{aligned}$$

where S is equal to the average number of detected photo-electrons per deposited keV in the active medium in the absence of ionization quenching. It is expressed in units keV$^{-1}$ and depends on the measurement geometry, on the scintillation yield and on the quantum efficiency of the PMTs.

The parameter *kB* depends solely on the scintillating material and should remain constant under different measurement conditions. When applying the Birks model, it is intriguing to fit equation (1) to the experimentally obtained light output data and attempt to estimate S and *kB* parameters. However, a challenge arises due to the strong correlation between these two parameters in a single measurement, leading to significant uncertainty in the estimates. To address this correlation issue, we conducted measurements by introducing neutral density filters between the LS (Liquid Scintillator) vial and PMTs (Photomultiplier Tubes), as described in^8^. This approach caused variations in light attenuation without altering the properties of the scintillator.

**Figure 2:**
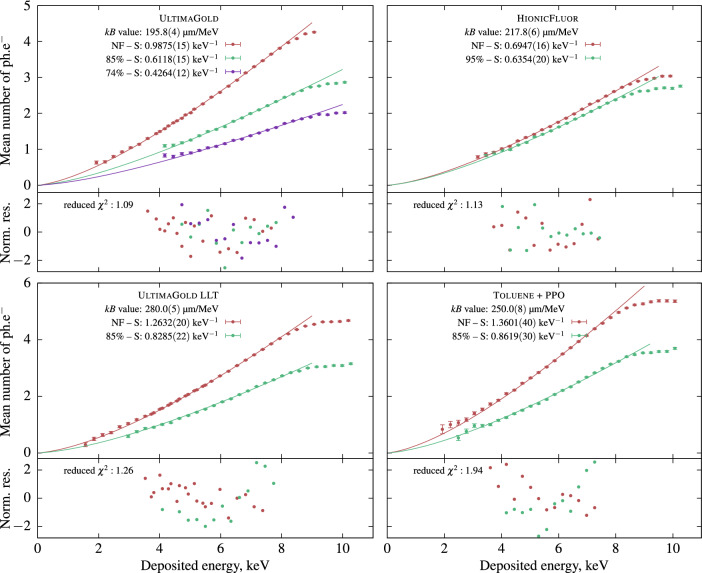
Mean number of detected photo-electrons ($\hbox{ph.e}^-$) as a function of the deposited energy for four samples in different LS cocktails. The first measurements were performed without neutral density filters (NF) and some of the samples were measured again with neutral density filters. The lines are the Birks’ ionization quenching formula with parameters shown in the legend. The normalized residuals are in units of standard deviations. The fits were conducted in the 2 to 8 keV range.

All LS samples, except for the HF cocktail, were measured both with and without an 85% transparent neutral density filter. The UG sample was measured with another filter with 74% transparency. The offline data analysis was performed using a 40 ns coincidence window and a 20 µs dead-time base duration, values chosen to match the timing performance of the liquid scintillation counter. The measurement results are presented in Fig. 2. All measurements were fitted using equation (2), with a shared *kB* parameter for all measurements conducted with and without neutral density filters. The S parameters were treated as free parameters in each measurement. We calculated the stopping power using the Bethe formula with the correction parameters provided by Tan and Xia^12^ for energies between 20 eV and 20 keV. The composition data for the commercial cocktails were obtained from the reference^13^ taking into account only the main organic compound of the scintillator.

The fits were obtained for the UG, UG LLT, and HF cocktails, resulting in reduced $\chi ^2$ statistic values ranging from 1.09 to 1.32. A higher reduced $\chi ^2$ value of 1.94 was observed for the toluene + PPO sample. The *kB* parameters obtained from the fit ranged from 195 µm$\cdot$MeV$^{-1}$ for Ultima Gold to 280 µm$\cdot$MeV$^{-1}$ for Ultima Gold LLT. While the shown method works in a rather narrow energy range, it is possible to extend it by replacing the $^{241}$Am source with a higher-energy emitter. Nevertheless, the non-linearity of scintillators is the strongest for these low energies that we have studied so in principle such range should be the most sensitive to the value of Birks’ constant

### Timing properties of liquid scintillation cocktails

**Figure 3:**
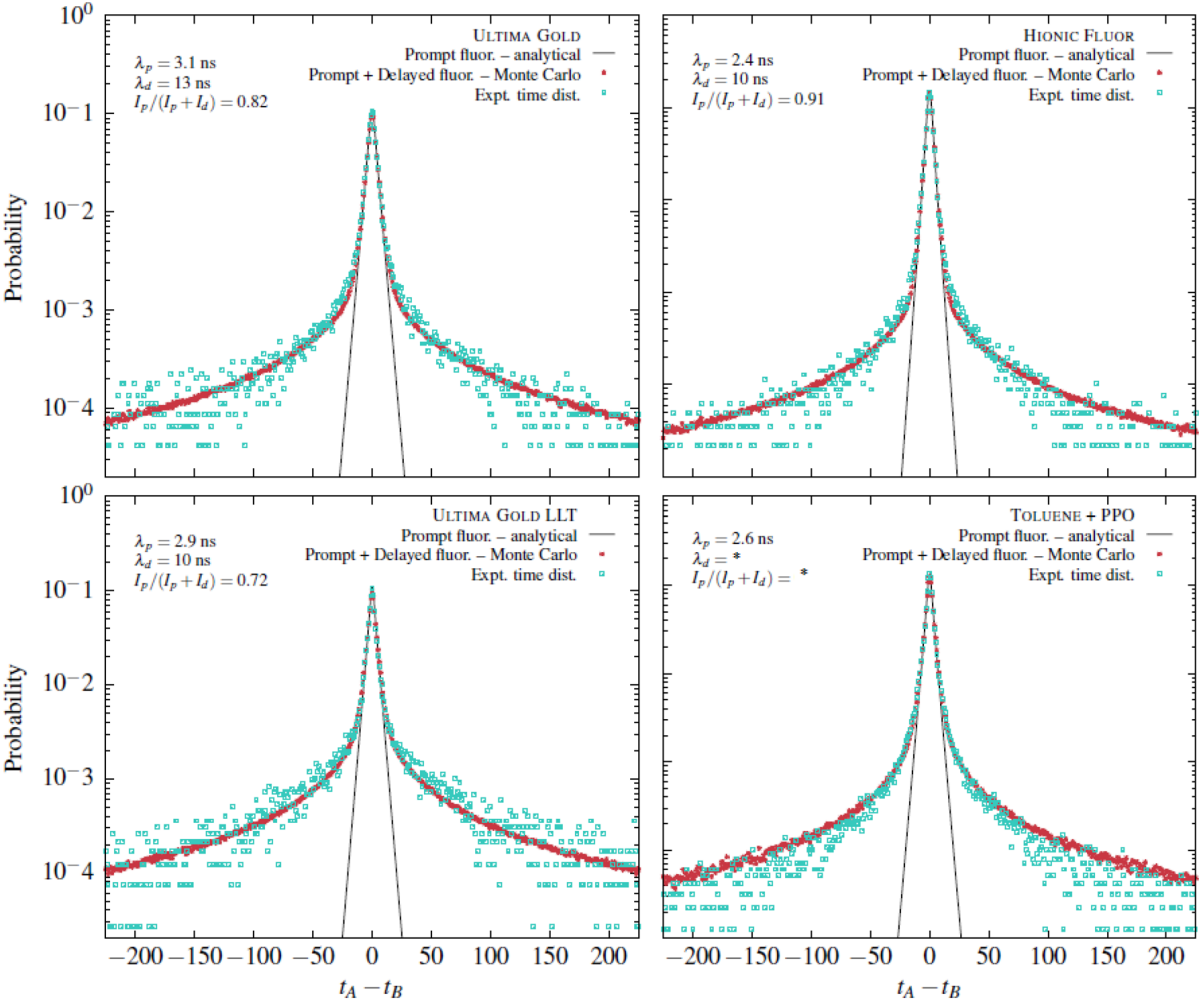
Time interval distribution of the four liquid scintillators cocktails at 5 keV. It is not possible to extract a slow component in the Tol-PPO scintillator, either because it does not exist or because we cannot measure it.

The timing properties measurement data for the four scintillating liquid cocktails, as described in 2.1, were processed using the method outlined in Characterization of the timing properties of scintillators and are depicted in Fig. 3.

With the experimental data and the analysis method, we can extract the time components $\lambda _p$ for the prompt fluorescence and $\lambda _d$ for the delayed fluorescence, as well as the proportion between the two, denoted as I$_p$ and I$_d$. The results are summarized in Table 1.

**Table 1:** Summary of the parameters measured for the liquid scintillation cocktails, we were not able to find a delay component value for toluene +PPO LSC, probably due to a limited sensitivity of our device.

Cocktail name	Relative light yield	$\lambda _p$, ns	$\lambda _d$, ns	$I_p/(I_p + I_d)$
Toluene + PPO	1	2.63 (26)	*	*
Ultima Gold	0.7265(24)	3.13 (31)	13.0 (13)	0.82 (8)
Ultima Gold LLT	0.9287(31)	2.91 (29)	10.0 (10)	0.72 (7)
Hionic Fluor	0.5340(19)	2.41 (24)	10.0 (10)	0.91 (9)

**Figure 4:**
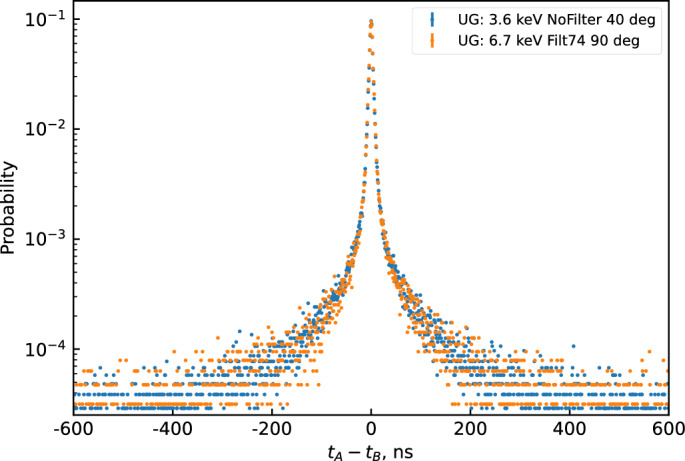
Time interval distribution at two different electron energies for the same average number of detected photons.

These results demonstrate our ability to accurately estimate the lifetimes of the fast and slow components, as well as their proportion, using these time distribution measurements and the corresponding analysis. An in-house developed Monte-Carlo code allows us to obtain the same distribution using the acquired data. We observe that the time components are very close for the three commercial scintillators. However, the proportion of the delayed component is more significant for UG LLT. This observation may help explain the findings from a previous study^14^, which suggested the need for a longer coincidence window in measurements involving such a scintillator compared to standard UG for low-energy radionuclides such as $^3$H. With a reduced coincidence window, some events may be lost, but the results are closer to Birks’ model, which does not take into account the delayed component of organic scintillators. Therefore, this measurement allows us to validate a model and optimize the use of instrumentation based on the model’s range of validity and the required accuracy. In a second step, we decided to utilize the capabilities of the device to analyze the temporal properties of scintillation for two different energies while keeping the photon count identical. These measurements were performed on a sample of UG in a PE-PTFE vial. To observe different energies, two measurements were conducted at incident beam angles of 40$^\circ$ (3.6 keV) and 90$^\circ$ (6.7 keV), with the photon count adjusted using neutral density filters. However, for these two different energies, the number of photons emitted by the scintillator was not the same. To observe the same number of photons, we employed a lab-made neutral density filter system developed for the TDCR device, as previously presented in a published work^8^. The experimental results are presented in Fig. 4.

The obtained results underscore our ability to perform measurements with precise energy selection and the potential to reduce the photon count without compromising the scintillator’s properties. Such adjustments may become necessary, given the sensitivity of the device in relation to the scintillator yield under analysis. Furthermore, these findings hold significant importance for the new opportunities arising from time distribution measurements in the field of radionuclide metrology, as previously discussed in related research^15^.

### Applications of the system for the characterization of plastic scintillators

The Compton-TDCR measurement device can also be utilized with plastic scintillators to investigate their response and optimize their chemical composition. In this context, we conducted a study on three solid scintillators developed at CEA. These three samples were shaped as comparable cylinders to match the geometry of a 10 mL liquid scintillator vial. The external surfaces of the scintillators scatter light in a manner similar to the plastic vial used with liquid scintillators. The production details of these scintillators are described in 5.4.

**Measurement of the light yield**

**Figure 5:**
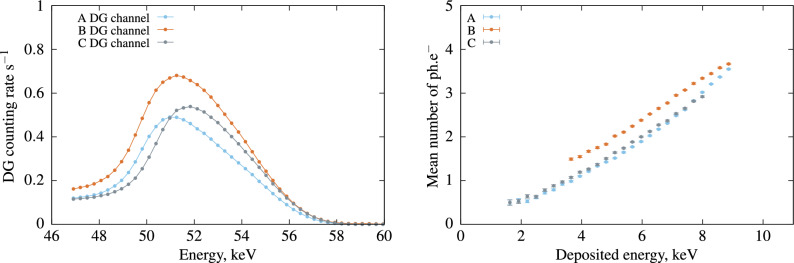
Left: Compton spectrum in the $\gamma$ channel for events in coincidence with D. DG correspond to the coincidences between the gamma channel of the CdTe (G) and the logical sum of the double coincidence in the PMTs (D). Right: Non-linearity curves showing the evolution of the mean number of photo-electrons as a function of the electron energy for the 3 plastic scintillators.

Figure 5 presents the results obtained for each plastic scintillator. Scintillators A and B were initially developed for their ability to discriminate between $\alpha$/$\beta$ and n/$\gamma$. They exhibit different non-linearity responses, with Scintillator A producing fewer photons than B. However, a notable difference between these two scintillators is observed in the low-energy region. By examining the Compton spectra, we can infer that their distinct compositions significantly impact the shape of the Compton spectrum, making the analysis at low energy more challenging. According to laboratory experiments involving measurements with $\gamma$ sources of different energies, Scintillator C was expected to produce more photo-electrons than the other two scintillators, A and B. Overall, its performance was estimated to be inferior, possibly due to the fact that the plastic piece was highly diffusive, resulting in a significant loss of photons at low beta energy. However, the advantage of this method lies not only in obtaining the scintillator’s performance but also in capturing the curve of non-linearity in the response, which demonstrates a significant difference between the samples.

**Figure 6:**
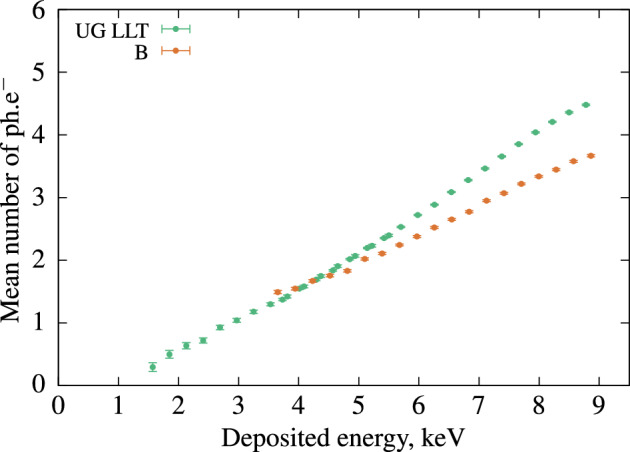
Non-linearity curves with the best plastic scintillator versus the best liquid scintillation cocktail.

Under 3 to 8.5 keV excitation, Scintillator B exhibited a higher scintillation yield than the other two scintillators (A and C). This method allows us to explore a different range of electron energy and reveals that between 1 keV and 9 keV, B dominates over the other tested samples. This result is crucial for the development of plastic scintillators, especially those designed for $\alpha$/$\beta$ discrimination, as it’s important to understand their response to low-energy electrons. Figure 6, based on the same dataset, demonstrates that plastic scintillators exhibit a significantly different non-linearity response compared to commercial LSC.

**Figure 7:**
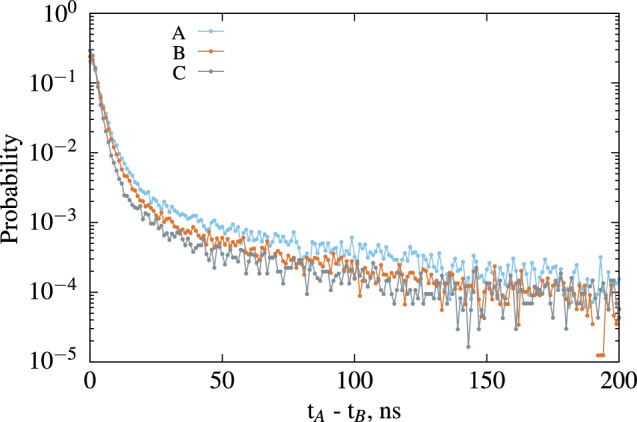
Time interval distribution of the three plastic scintillators for same number of ph$$\cdot$$$$\hbox{e}^{-}$$, the corresponding energy is 7 keV for A and C and 6.2 keV for B.

**Measurement of the timing properties** The data from each Compton measurement with the three plastic scintillators were reprocessed to obtain the timing properties for the same average number of photons, as shown in Fig. 7.

The extracted timing properties of these scintillators are presented in Table 2.

**Table 2:** Summary of the parameters measured for the 3 plastic scintillator samples.

Cocktail name	$\lambda _p$, ns	$\lambda _d$, ns	$I_p/(I_p + I_d)$
A	3.62 (36)	15.1 (15)	0.83 (8)
B	3.17 (31)	11.8 (12)	0.83 (8)
C	2.54 (25)	9.80 (10)	0.91 (9)

As demonstrated here, it is possible to extract this information from either a liquid or plastic organic scintillator. Therefore, it would be feasible to use a liquid formulation that matches the composition of the plastic scintillator just before its polymerization. This approach would not only allow us to investigate the distinctions between the two solid and liquid media and their resulting scintillator properties but also eliminate the need for the complex polymerization step, enabling the exploration of scintillator formulations at an earlier stage. In this study, we compare the toluene+PPO liquid scintillation cocktail to the plastic sample C, with the only difference being the organic solvent; the dye compounds are the same. For samples C and Toluene+PPO, the variation in light output between the two scintillators can be directly attributed to charge transfer within the medium. In the liquid, the molecules have greater freedom of movement compared to the plastic, resulting in a slower response time for the plastic, characterized by a larger slow component of approximately 10 ns, while it was not possible for us to assess a slow component in the toluene based liquid mixture. However, it’s important to consider the structure of $\pi$-electrons, which could potentially differ before and after polymerization. This comparison might pose challenges, but at the very least, it allows us to identify the potential impact of this effect.

### Applications of the system for the characterization of inorganic crystal scintillators

Inorganic scintillators typically possess significantly higher density and Z$_{eff}$ values compared to organic and liquid scintillators. As a result, they exhibit much greater X-ray or $\gamma$ photon absorption capacity, higher photoelectric absorption cross-sections, and lower Compton absorption cross-sections. Their scintillation efficiency can reach up to 100 ph$\cdot$e$^-$ $\cdot$keV^16^. Due to their high density and Z$_{eff}$, the crystal’s geometry must be carefully adjusted to prevent the re-absorption of scattered $\gamma$ photons in the Compton-TDCR experiment, which operates at relatively low energy.

For this experiment, we used the well-known scintillating material Y$_3$Al$_5$O$_{12}$:Ce$^{3+}$ (YAG:Ce) with a density of 4.56 g$\cdot$cm$^{-3}$. It was provided in the form of a single crystal fiber produced using the micro-pulling down technique^17^. This 2 mm diameter and 20 mm long fiber was placed at the center of a PE-PTFE tube within a PE-PTFE liquid scintillation vial.

**Measurement of the light yield**

**Figure 8:**
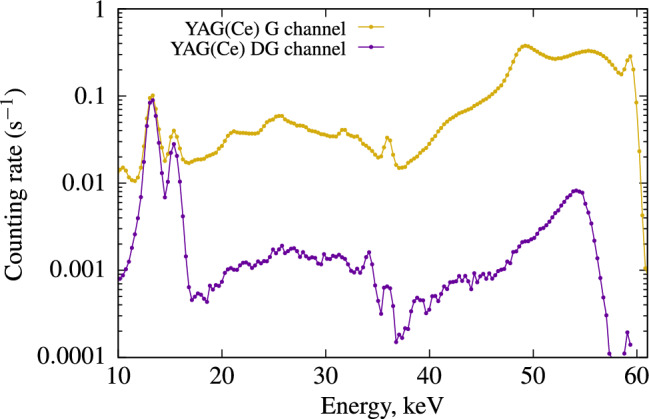
Compton spectra in the $\gamma$ channel for event with and without DG coincidence.

Thanks to its specific shape, it is possible to obtain a Compton spectrum in the $\gamma$ detector in coincidence with the photons detected in the photomultiplier. This spectrum is presented across the entire energy range in Fig. 8.

**Figure 9:**
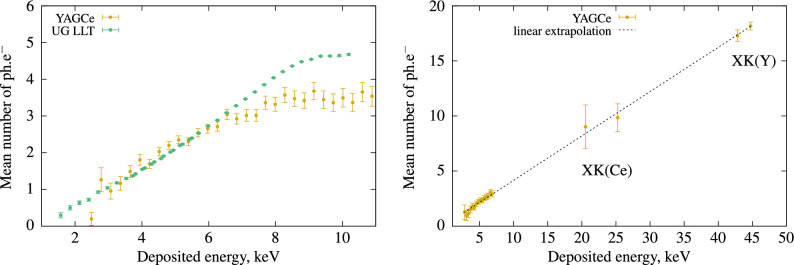
Left: Non proportionality response of YAG(Ce) single crystal compared to the best commercial liquid scintillator (UG LLT). Right: results with extended energy using X-Rays fluorescence data.

However, we observe a significant difference between the two spectra, requiring long measurements to collect a sufficient number of photons in DG coincidence. This difference is primarily due to the low probability of Compton scattering in this material, as photoelectric absorption dominates at the low energy levels used in this study (59.54 keV). Nevertheless, from this Compton spectrum, we can deduce the non-linearity response at low energy for the YAG(Ce) scintillator, and the results are presented in Fig. 9.

Compared to plastic scintillators, YAG(Ce) is quite close to the commercial LSC, UG LLT. However, the shape and refractive index are significantly different, affecting light collection. Unlike organic scintillators, due to the small size of the fiber, some of the photons from $^{241}$Am interact, and the peak of $^{241}$Am at 59 keV disrupts the Compton spectrum. Consequently, it is not possible to analyze the data corresponding to Compton electrons at energies below 3 keV. At higher Compton electron energies, around 7 keV, the results are unexpectedly lower than those for the liquid scintillator. Another factor to consider is that YAG(Ce) has a peak emission at a wavelength of 550 nm, unlike UG LLT, which is located at the quantum yield peak of our PMT at 400 nm. For YAG(Ce), the quantum yield of PMTs is three times lower than that of UG LLT.

The heavy elements (Yttrium and Cerium) in the inorganic crystal exhibit a relatively intense X-ray fluorescence probability, with clearly detectable peaks that are highly interesting to analyse. As shown in Fig. 8, it is possible to observe the XK$\alpha$ and XK$\beta$ emissions of Yttrium between 14 and 17 keV, as well as the XK$\alpha$ and XK$\beta$ emissions of Cerium at approximately 34 keV and 39 keV, coinciding with the light photons detected in the Compton device. Since the detection of scintillation photons is exclusively analysed in coincidence with X-ray fluorescence originating from the K levels, there is no contribution from hole relaxation in the K bands to light production. This information allows us to extend the energy range of purely electronic excitation to include photo-electrons originating from the K-bands of Y and Ce. The X-rays emitted from the materials result from internal recombination in these atoms, and the light produced in coincidence with these X-rays is due to electrons with an energy E$_e$ = 59.54 - E$_X$. These results are presented in Fig. 9, with some additional data points at higher electron energies. Given that the cerium element is a doping ion, its concentration is low, and there is greater uncertainty in the Ce X-ray measurements due to limited counting statistics during the measurement period. Various techniques have been proposed to analyse the non-proportionality of inorganic scintillators. The most common approach involves conducting pulse height spectra measurements with different radioactive sources emitting at various $\gamma$ energies^18^. An alternative method employs synchrotron radiation, where the crystal is excited by monochromatic X-rays with variable energy. In this case, significant non-proportionality is observed near the X-ray absorption thresholds^19^. In both of these techniques, the light produced originates from photo-electrons as well as auger relaxation of holes generated in the core levels. The method closest to the one presented in this article was proposed by^2^. It involves measuring the light produced by an electron resulting from Compton scattering of a $\gamma$ photon at 662 keV using pulse height spectra. A similar experiment, named SLYNCI (Scintillator Light-Yield Non-proportionality Characterization Instrument), was conducted more recently with the implementation of five High Purity Germanium (HPGe) $\gamma$ detectors to enhance measurement statistics^20^.

**Figure 10:**
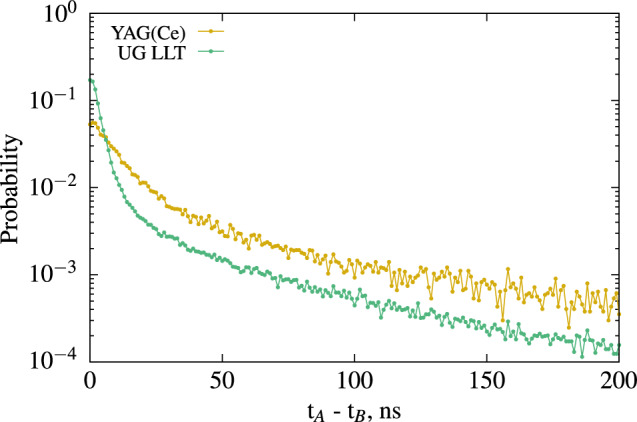
Time interval distribution between PMT-A and PMT-B of the YAG(Ce) sicntillators compared the LSC UG LLT at 6 keV electron energy for the same average number of photo-electrons.

**Measurement of the timing properties** The scintillation mechanism in inorganic scintillators is generally described as a three-stage process: creation of electron–hole pairs and their thermalisation, migration and energy transfer to the luminescent centre, and luminescence. During the second stage, migration and traps can induce significant changes in the timing properties compared to direct optical excitation^21^. Determining the timing properties is therefore crucial for applications using coincidence techniques or time of flight measurements. YAG:Ce crystals typically exhibit a slow component. Since the scintillator yields approximately the same output as UG LLT at 6 keV, we can directly compare the timing distribution results, as shown in Fig. 10, which reveal significant timing characteristic differences from UG LLT.

**Figure 11:**
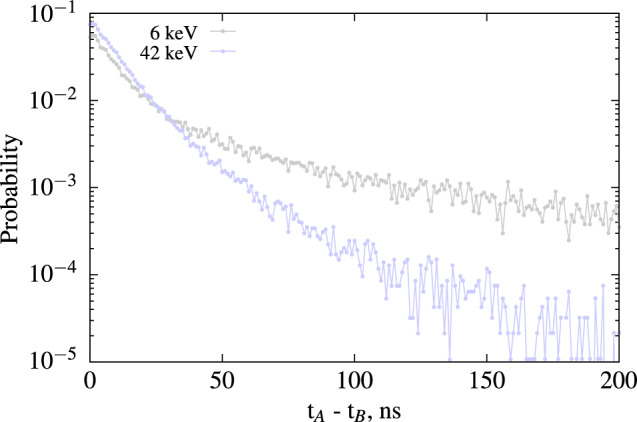
Time interval distribution of photon produces by YAG(Ce) for an energy of 42 and 6 keV.

We can also extract the timing distribution for various energy ranges, as illustrated in Fig. 11, using the X-ray peak as a $\gamma$ gate. This enables us to extract the intrinsic timing properties of the scintillator using the method described in Sect. Characterization of the timing properties of scintillators. Unlike the time interval distribution of the first two photons, the latter refers to the scintillation decay time and is independent of the number of detected photons. Any potential changes with electron excitation energy are solely due to variations in the scintillation mechanisms resulting from fluctuations in ionization density.

From the measured data using the method presented in Sect. Characterization of the timing properties of scintillators, we can conclude that this YAG(Ce) crystal, when interacting with electrons, exhibits a fast component of 11 ns at 6 keV and 11.2 ns at 43 keV, along with a second component of 78 ns at 6 keV and 69.0 ns at 43 keV. At 6 keV, the fast component represents 85% of the collected light, and at 43 keV, it accounts for 90.3%. The higher proportion of the slow component for low energies is consistent with theory, as it corresponds to a smaller amount of light being produced. Due to the low counting statistics, we estimate the time constant of the second component with a relative standard uncertainty of 10%, and the results are consistent for both analyzed energies.

## Discussion

In this paper, we have presented the design of an ultra-sensitive and precise photon counting instrument for characterizing the response of various types of scintillators to low-energy electrons. We have explained and detailed the system’s different capabilities. This instrument not only allows us to measure the scintillation efficiency for different electron energies but also provides insights into the scintillator’s timing properties. Specifically, we have demonstrated the ability to deduce the timing properties of both the slow and delayed components of organic scintillators, along with their respective proportions. Furthermore, we have shown that this device can extract valuable information related to plastic scintillators, similar to how it operates with liquid scintillators. It offers a distinct advantage by enabling the study of response differences between the two materials with similar compositions. The instrument’s sensitivity facilitates the examination of variations in scintillation efficiency related to charge mobility within the scintillator. This mobility differs between liquids and solids and can be estimated by analyzing the non-linearity of the response to low-energy electrons ranging from 2 to 8.5 keV. This initial demonstration underscores the potential of such instrumentation and introduces one of the pioneering approaches in this field. Future developments may include Monte Carlo modeling of the device, optimizing its design, and enhancing our understanding of scintillation models by introducing precise ionization interaction modeling. This instrumentation has also allowed us to measure the response of inorganic scintillators, made possible by tailored shaping of the scintillator in a capillary form. Typically, inorganic scintillator responses are measured through the photoelectric peak, but our analysis of X-ray fluorescence from the material enabled a comprehensive study of YAG’s response from 3 to 43 keV. The current instrument’s energy measurement capacity is limited, but this can be easily addressed by using other mono-energetic sources, such as $^{99m}$Tc (140 keV), $^{139}$Ce (166 keV), $^{51}$Cr (320 keV), or $^{137}$Cs (661 keV), to generate higher energy beams. As a portable instrument, it can be conveniently relocated to various beam sources, including those induced by synchrotron X-rays, Compton-induced sources, or X-ray tube-filtered sources. Moreover, its applicability can be extended to other types of particle beam sources. This makes it a valuable tool for our future research on innovative and complex scintillators. Implementing such a source will require modifications to the shielding, which can be optimized through Monte Carlo simulations of the system. Additionally, the gamma detection efficiency can be improved by replacing the CdTe detectors with three HPGe detectors positioned between each photomultiplier tube, resulting in a twofold enhancement in energy resolution.

## Conclusions

The significance of this instrumentation spans multiple dimensions and aligns with various objectives. Firstly, we have demonstrated the feasibility of such an approach by detailing all the methodologies used and comparing them with some of the existing models to identify any potential differences. We have also proposed solutions to extend the energy range, aiming to achieve a higher level of precision on a broader scale. This expansion will enable us to utilize the instrument in the future for radionuclide metrology. The compact size of the device allows for easy mobility, enabling its use in diverse settings, such as in a photon beam from a synchrotron, to attain precise energy measurements. Secondly, this instrument contributes to characterizing the response of well-established scintillators and enhances our comprehension of emerging scintillation models currently under development^10^. The capability to measure the responses of diverse scintillator types, as demonstrated in our work, holds great promise for model comparisons, thereby advancing our understanding of the underlying phenomena. Lastly, this instrument holds particular relevance for novel scintillators with unknown properties, such as porous scintillators developed within the framework of the SPARTE project^1^. It is already being utilized for these innovative materials, offering valuable insights and applications beyond traditional scintillators.

## Methods

### Compton coincidences system design

**Figure 12:**
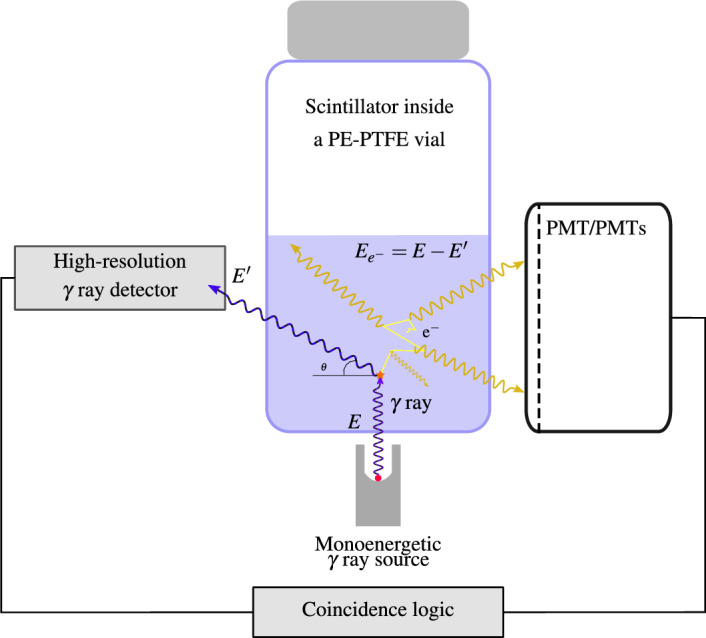
Schematic representation of a Compton coincidences system. The energy of the electron that is deposited in the cocktail can be determined from the energy of the scattered $\gamma$ ray.

The development of a Compton coincidence measurement system involves the utilization of an external collimated mono-energetic $\gamma$-ray source, as depicted in Fig. 12. This source directly interacts with an energy denoted as *E* within the scintillator. A $\gamma$-ray detector positioned at a specific angle relative to the source beam is used to measure the Compton-scattered photons with energy *E’* within the scintillator. When these scattered photons are detected in coincidence with the light photons emitted by the scintillator, we can derive the scintillation efficiency as a function of the energy of the interacting electrons, denoted as *E* $_{e-}$.

**Design** The design of the detector was developed in conjunction with the development of the miniTDCR device at LNE-LNHB^8^. Several considerations and compromises were made in accordance with the requirements of the previously described method concept:

- The Light Photon Counter: We opted for a portable TDCR device equipped with three photomultiplier tubes (PMTs) known for their high efficiency. This configuration enables the accurate measurement of light photons and is capable of accommodating standard 20 mL Liquid Scintillation vials, corresponding to cylinders with a maximum diameter of 25 mm and a height of 60 mm. Such a counter, utilizing the TDCR method, ensures precise light photon measurements compared to devices with only one or two PMTs.
- The Gamma Detector: It had to be compact, possess good detection efficiency, and provide high resolution. It was strategically positioned between two of the PMTs and placed as close as possible to the liquid scintillation vial to enhance geometrical efficiency. While germanium detectors are often considered ideal, they come with limitations in terms of price and size, mainly due to the required cooling systems. In this project, we selected a CdTe detector, the XR-100T from Amptek^22^, which employs a compact thermoelectric cooling cell. This detector boasts dimensions of 25 mm$^2$ with a 1 mm-thick CdTe layer and utilizes a specific signal shaping technique known as Rise Time Discrimination (RTD)^22^ to enhance resolution, albeit at the expense of longer pulse timing. The CdTe detector was chosen as a compromise to optimize the solid angle between the sample and the detector. Small-sized HPGe detectors were no longer available, and a larger detector would have required adjustments to the sample-to-PMT distance, potentially diminishing the TDCR efficiency.
- The Mono-Energetic Beam: The mono-energetic beam was generated using a sealed source containing 74 MBq of $^{241}$Am. This source was housed in an A3015 capsule with a stainless steel shell, which already provided effective shielding for the primary 59.54 keV line, especially on the side and bottom of the source. This configuration produced a filtered beam on the top of the source, eliminating low-energy peaks. The source holder was designed to be opened or closed and could be adjusted to alter the angle of beam interaction with the vial while maintaining the interaction point at the center of the vial.

**Figure 13:**
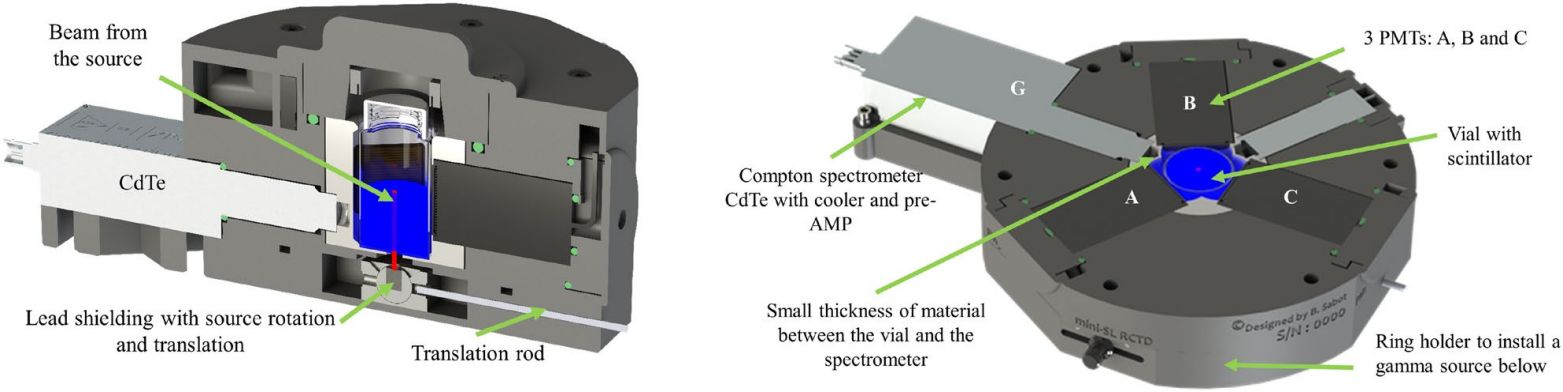
Left: Vertical cut of the system—the red line represents the beam of the source, the bright blue correspond to the size of scintillator ; Right: Horizontal cut of the system at the position of the CdTe detector.

Figure 13 depicts a horizontal cross-section of the designed device. The device is equipped with three R7600U-200 PMTs from Hamamatsu, each operating at a positive high voltage. Utilizing Fused Deposition Material (FDM) 3D printing technology, we were able to create complex components, particularly optimizing the optical chamber to achieve a high detection efficiency. The 3D printing method also allowed for the inclusion of recessed sections in some parts, minimizing the absorption of $\gamma$-rays between the LS vial and the gamma detector, as shown in Fig. 13.

Additionally, the device incorporates a filter holder in the cap to reduce the number of photons originating from the vial that reach the PMTs. Various levels of neutral density filters can be employed with this holder. The source holder module, situated beneath the scintillation counter, is designed to be easily removable. This design feature enables straightforward replacements to accommodate different types of single-energy sources, facilitating studies at varying beam energies.

**Figure 14:**
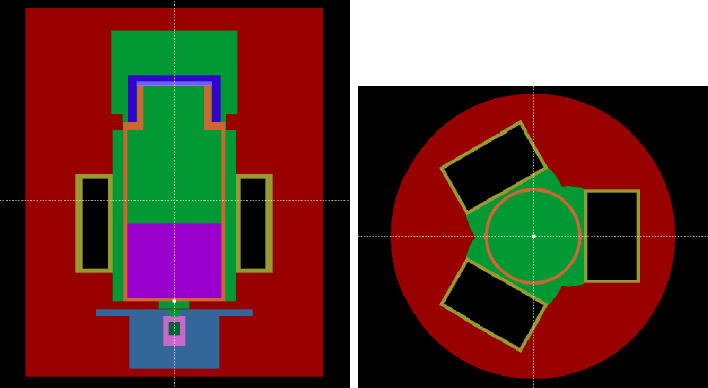
Two cut of the Monte-Carlo model built for PENELOPE 2018. The colors represents the different materials that compose the device: RED is the 3D printed device made of PLA, bright green is air, black is the vacuum of PMTs, yellow is the quartz of the PMTs, orange is the PET from the vial, purple is the scintillator (UG), grey is lead shielding of the source, pink is the source shielding made of stainless steel 304, dark green is the $^{241}$Am source mainly composed of ceramic.

**Monte Carlo Simulations** In order to simulate the absorbed energy in the scintillator and the source holder design, a Monte-Carlo model of the TDCR device was adapted to include the properties of the source holder and the $^{241}$Am source itself. A visual cross-section of the Monte-Carlo model is presented in Fig. 14. The model is created using the Penelope 2018^23^ Monte-Carlo code, which was chosen due to its detailed transport model for low-energy photons and electrons. It also allows us to directly incorporate the decay scheme of a radionuclide for simulations, in this case, $^{241}$Am.

**Figure 15:**
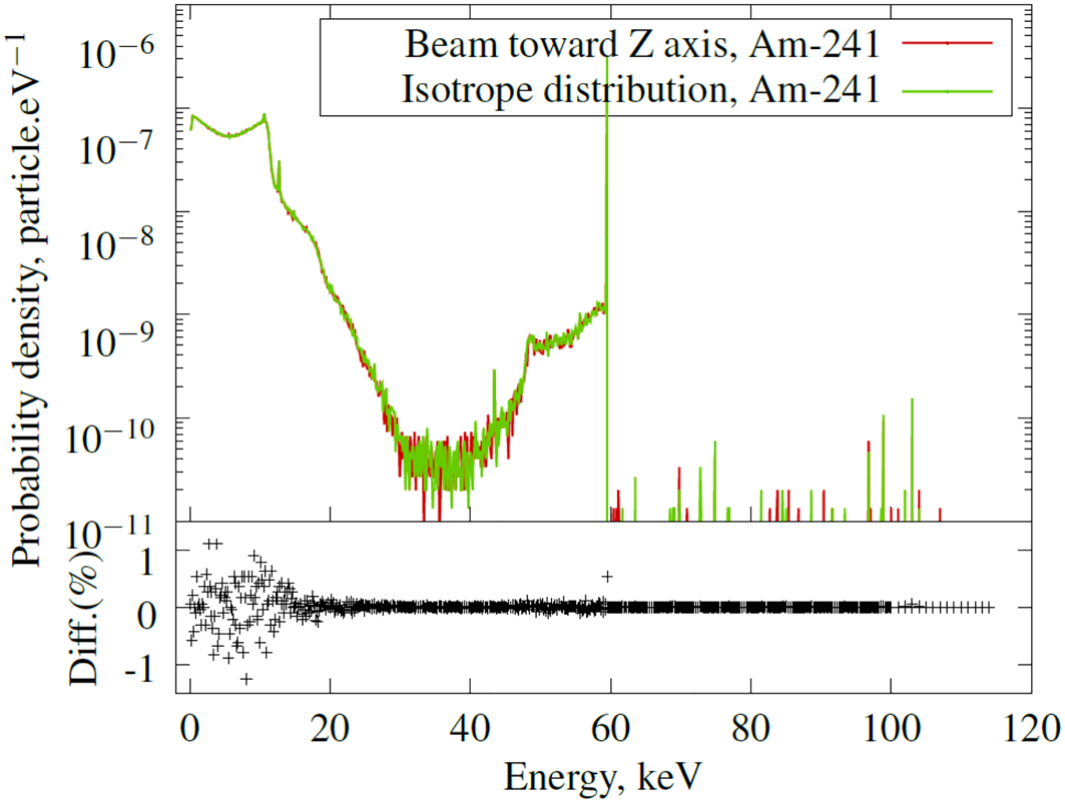
Results of simulated absorbed spectra from $^{241}$Am inside the commercial liquid scintillator UG.

To observe the effect of the shielding on the shape of the absorbed spectra within the liquid scintillator, a simulation was performed using UG scintillator properties^13^. The simulation results are presented in Fig. 15, demonstrating the main absorption of the 59.54 keV line from $^{241}$Am (with an intensity of 35.92%) and the Compton spectra in the scintillator.

Two peaks are visible in the low-energy range: one at 10.6 keV and one at 12.7 keV, corresponding to the XL line of Pb from the shielding. To eliminate these small energy peaks, a 1 mm layer of aluminum can be used, resulting in a slight 1.5 keV XK line (fluorescence efficiency of 4%) that could be absorbed by the plastic layer and the liquid scintillation vial. Such a shield would reduce the 59.54 keV beam intensity by only 7%. In this case, aluminum is the best choice, while our first idea of iron or copper produce 7 and 8 keV XK lines with 30% and 40% fluorescence efficiency, respectively, which are not entirely absorbed by the materials present along the beam path before reaching the scintillator.

These simulations allow us to study the influence of all the gamma emissions from the $^{241}$Am source, especially the contribution of low-probability but higher-energy peaks compared to 59.54 keV. The contribution of energies higher than 59.54 keV represents only 0.03% of the total surface of the absorbed spectra in the scintillator, which is negligible (see Fig. 15). However, in the low-energy region, when comparing a simulated mono-energetic beam of 59.54 keV with 5.92% emission to the $^{241}$Am decay scheme, the difference is 3.2%, highlighting the importance of simulating the decay scheme instead of relying on a simple mono-energetic beam. This difference is due to the higher-energy emissions, which deposit some scattered energy, as seen in the 20 keV to 40 keV region in Fig. 15, that are not absorbed by the shielding of the source’s window.

The final simulation assumed that the $^{241}$Am source only produces a beam in the Z-axis (as shown in Fig. 14) in the direction of the vial. The results were compared to simulations performed with an isotropic distribution (as seen in Fig. 15), resulting in a difference of 0.19% across the entire spectra surface, with no noticeable changes. This confirms that the system is designed to produce a proper beam that enters the vial and deposits energy into the liquid or solid scintillator.

**Energy calibration and resolution**

**Figure 16:**
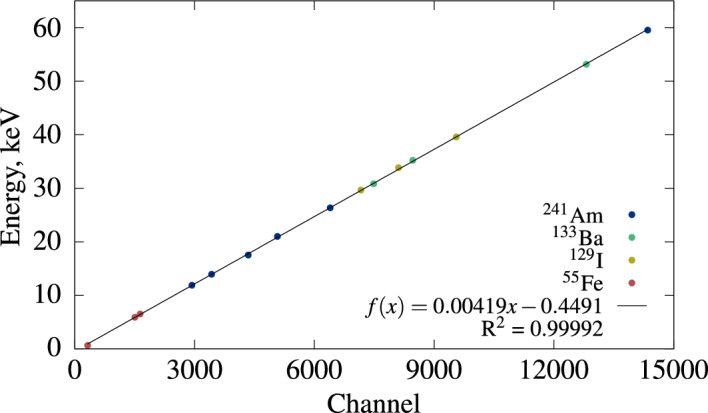
Energy calibration of the CdTe detector with spectrometric $\gamma$ sources.

The CdTe detector was connected with the CAEN digitizer and calibrated using 15 lines from four punctual spectrometric sources produced at CEA/LNHB: $^{241}$Am, $^{133}$Ba, $^{129}$I, and $^{55}$Fe. The energy calibration is visible in Fig. 16, where the data was fitted with a linear function. The detector exhibits very good linearity in the range from 0.6 keV to 60 keV. With the current acquisition setup, this detector achieves a resolution between 350 and 500 eV in this same energy range.

### Measurement data analysis for the scintillator non-proportionality

**Measurement data processing and accidental coincidences correction:**For each measurement, the CAEN digitizer outputs *list-mode* files containing timestamps and the deposited energy in the detector. These files are processed using an in-house-developed software that applies standard dead-time logic with user-defined coincidence windows and dead-time base duration. The code calculates the single and coincidence counting rates in all coincidence channels, between PMT A, B or C and each energy bin in the G channel which is the $\gamma$ detector (AG, ..., ABG, ..., ABCG, DG). The offline data analysis, along with the developed code, provides the capability to determine the relative light output of the scintillator as a function of energy for arbitrarily long coincidence resolving times, all with a single measurement.

To achieve reasonably high counting rate coincidences with the $\gamma$ channel, it is necessary for the activity of the external source and, consequently, the counting rate in the scintillator sample to be relatively high. In the presented system, typical counting rates in the single PMT channels range from 5000 s$^{-1}$ to 12000 s$^{-1}$. Therefore, corrections for accidental coincidences are necessary, particularly when studying long coincidence resolving times. The correction for accidental coincidences in the presented studies was performed using the experimental method described in reference^9^. The true counting rate $N_t^{(i)}(\tau )$ in a coincidence channel *i* for a coincidence window $\tau$ is calculated as:

3 $$\begin{aligned} N_t^{(i)}(\tau ) = N^{(i)}_m(\tau ) - \left( \frac{N^{(i)}_m(2500 \text { ns}) - N^{(i)}_m(2000 \text { ns})}{500 \text { ns}}\right) \tau , \end{aligned}$$

$N_m^{(i)}$ represents the measured coincidence counting rate in channel *i*. The underlying assumption is that for coincidence windows exceeding 2000 ns, any increase in the counting rate with a larger coincidence window is primarily due to accidental coincidences. Moreover, it is assumed that the distribution of accidental coincidences can be approximated as a linear function. The mean number of detected photons, denoted as ${\bar{n}}$, in a given PMT for a specific energy *E* is calculated using the equation derived in reference^24^:

4 $$\begin{aligned} {\bar{n}}_X(E) = -3 \ln \left( 1 - \frac{TG(E)}{YZG(E)} \right) ,\quad X = (A, B, C),\ YZ = (AB, BC, AC),\ X \ne Y \text { or } Z. \end{aligned}$$

Here, *TG* and *YZG* are the counting rates in the respective coincidence channels coinciding with the $\gamma$-detector at a certain energy *E*.

Equation (4) applies to mono-energetic events. However, in practical applications, a narrow energy gate must be employed in the $\gamma$ channel. If the energy gate width is sufficiently small, Eq. (4) remains valid. In the present studies, the width of a channel of the $\gamma$-detector is 270 eV, which is considered narrow enough for the mono-energetic approximation. Another assumption made to apply Eq. (4) is that the three PMTs of the detection system are independent. This assumption may not hold when using clear glass vials. However, for all the studies presented, PTFE-coated PE vials were used, which are diffusive and significantly scatter the emitted light from the cocktail. The mean number of detected photo-electrons in all three PMTs is obtained by summing the mean number of detected photo-electrons in each PMT:

5 $$\begin{aligned} {\bar{n}} = {\bar{n}}_A + {\bar{n}}_B + {\bar{n}}_C. \end{aligned}$$

**Compton spectra analysis energy range**

**Figure 17:**
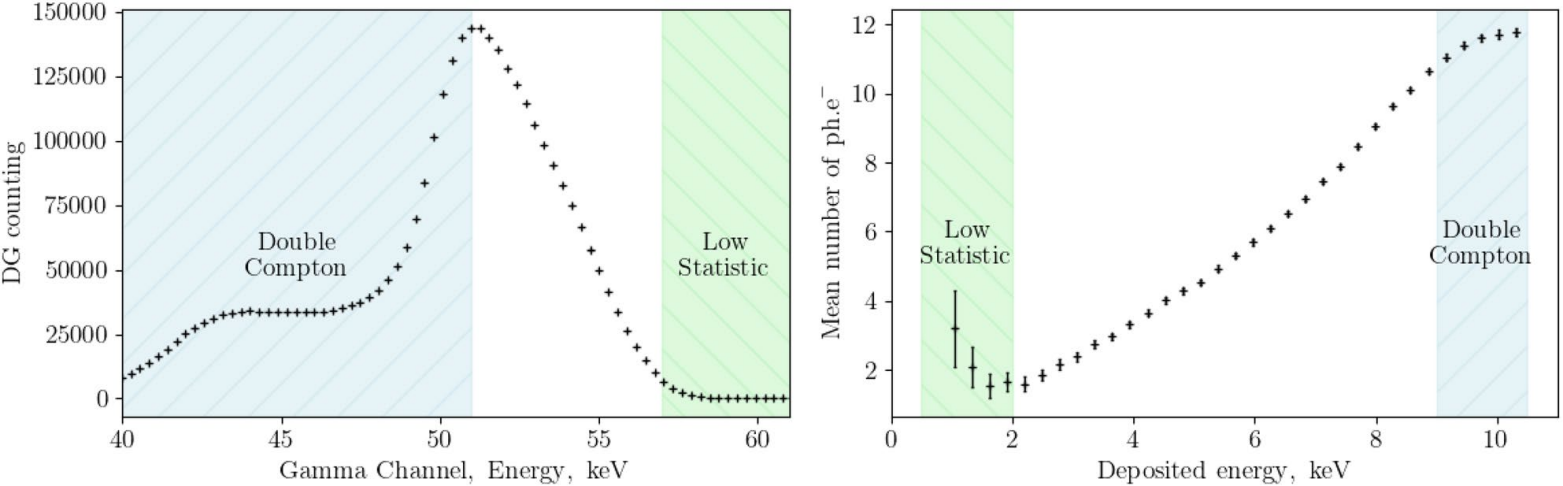
On the left, the obtanied compton spectra in coicidence with D channel, on the right the extracted non-linearity curve. The boundaries are represented in colors on the pictures to illustrate our current limitations.

The presence of subsequent Compton scatters depends on the energy deposited by the first scatter, impacting our ability to accurately estimate the number of photons produced in relation to the deposited energy. As illustrates in the Fig. 17 at higher energy levels, beyond a certain threshold, we encounter challenges due to the presence of double Compton scattering events, which can lead to inaccurate estimations limiting our capability to analyse at energies higher than 8.5 keV.

Furthermore, as we approach the $^{241}$Am peak, in cases where only a few photoelectric events occur, measurements can become unreliable. This limitation typically occurs around the 2 keV threshold but can be even larger for inorganic scintillators. Additionally, investigating Compton electron energies that result in an average detection of less than 1 photo-electron is impractical due to the extremely low counting rates observed in coincidences between the TDCR and $\gamma$ detectors.

**Figure 18:**
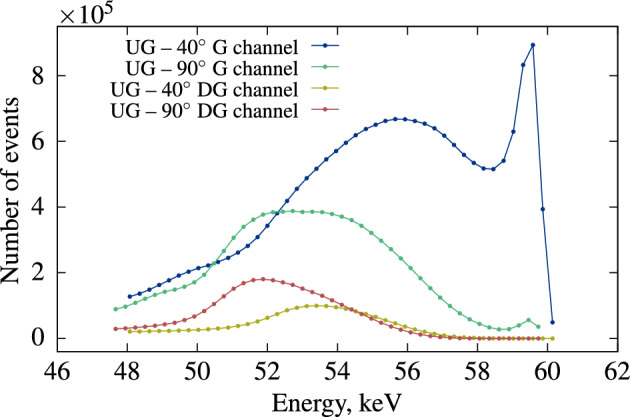
Energy spectrum in the $\gamma$ channel from the CdTe without (G) and with (DG) coincidences with the D channel of the TDCR detector for the 40$^\circ$ and 90$^\circ$ angles. The $\gamma$ beam is produced by the collimated $^{241}$Am source.

**Variable angle validation** The external source holder is designed to facilitate horizontal translation and rotation, allowing for adjustments in the angle between the collimated source and the exact center of the scintillator. Changing this angle enables the selection of Compton electron energies with the highest probability of detection in the $\gamma$ channel. Figure 18 displays spectra from the CdTe detector, both without and with coincidences with the D channel of the TDCR detector, at two different angles between the source beam and the CdTe detector.

**Figure 19:**
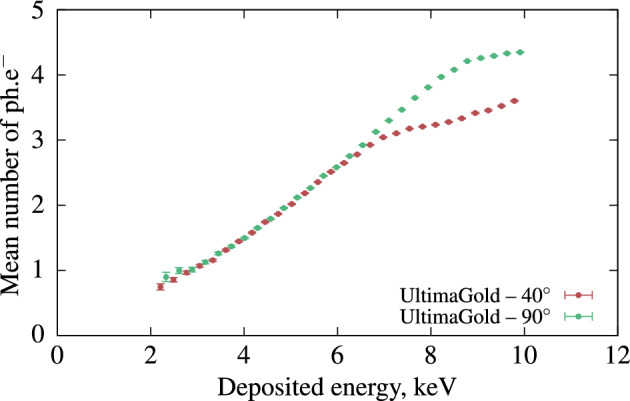
Relative light output of 10 mL Ultima Gold in PE-PTFE coated vial measured at 40$^\circ$ and 90$^\circ$ between the source and detector.

At a 40$^\circ$ angle, the Compton peak shifts towards higher energies, increasing the likelihood of detecting low-energy Compton electrons in the sample (as illustrated in Fig. 12). An important consideration in Compton coincidence systems is the potential asymmetry of the PMTs, which can result from one PMT being directly irradiated by the source. A similar bias may occur if a significant portion of interactions in the LS takes place far from the center of the vial. Such biases are sensitive to the source’s position relative to the measured sample and the angle between the source and the detector. In a well-optimized system, the measured light output for a given energy should remain consistent across different source positions and angles. Figure 19 depicts two measurements of the relative light output at two different angles between the source and detector. At 90$^\circ$, the external source is positioned directly below the center of the vial, while at 40$^\circ$, it is located 1 cm off-center, with the beam maintained in the middle of the vial. The light output obtained from these two measurements closely aligns in the energy range of 3 to 6 keV deposited energy.

However, at 40$^\circ$, the likelihood of multiple Compton scattering events becomes higher above 6 keV, making it impractical to measure light output. Conversely, below 3 keV, the results are more accurate, with lower uncertainty due to higher counting statistics. At energies exceeding 6 keV, the probability of small-angle scattering is significantly lower at 90$^\circ$ compared to 40$^\circ$, resulting in the divergence seen in Fig. 19.

The agreement between the results from the two measurement geometries indicates that the system is not biased toward a particular PMT and allows for the determination of the accurate energy range for this method.

### Characterization of the timing properties of scintillators

**Figure 20:**
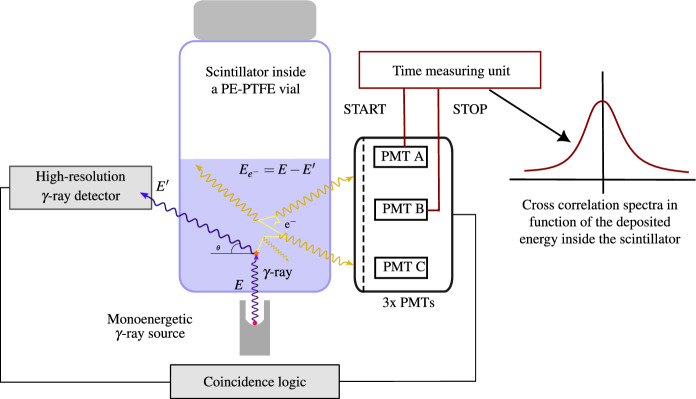
A schematic illustration of the acquisition of the cross-correlation distribution in the Compton spectrometer.

**Principe**The Compton-TDCR system allows us to access precisely the number of photons as a function of the energy deposited in the scintillator. The entire coincidence process allows for detailed data analysis, which can be further refined through list-mode data. As illustrated in Fig. 20, it becomes feasible to measure the time distribution of light photons between two PMTs, namely A and B, for a gamma energy detected in the gamma detector. Subsequently, the information gleaned from the time distribution results must be processed, as outlined below.

**Models and assumptions**

The intensity of fluorescence in a single-component scintillator decreases exponentially with time, following the law^11^:

6 $$\begin{aligned} I_p = I_{p0} e^{-t/\lambda _p}, \end{aligned}$$

**Figure 21:**
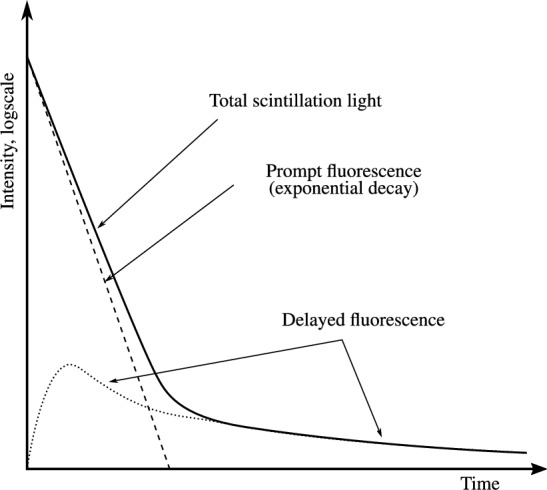
Schematic representation of the prompt and delayed emissions to the total scintillation light. Figure adapted from^25^.

Where $I_{p0}$ is the initial light output at time $t = 0$, and $\lambda _p$ is the fluorescence lifetime. The time dependence of the scintillation intensity of delayed fluorescence is significantly more complex than that of the prompt component, as it is influenced by the diffusion process of triplet states. A schematic representation of the time dependence of the two scintillation components is shown in Fig. 21.

One of the most comprehensive attempts to quantify the intensity of the delayed component over time, denoted as $I_d(t)$, can be found in the work of King and Voltz^26^. In this work, the time dependence of delayed fluorescence is modeled using a diffusion kinetic equation that describes the local density of delayed singlet states, represented as $c'_s (r, t)$:

7 $$\begin{aligned} \frac{\delta c'_s(r,t)}{\delta t} = D_s \nabla ^2_r c'_s (r, t) - \frac{1}{\tau _s}c'_s(r, t) + k_u c^2_T(r, t), \end{aligned}$$

The term $k_u c^2_T(r, t)$ in the equation describes the production of delayed singlet states through the bimolecular interaction between two $T_1$ states. The equation is solved with the initial condition that $c'_s(r, 0) = 0$. The initial distribution of the triplet states is derived for two cases: one for particles with low stopping power, where ionisations are far apart and do not interact, and the other for particles with high linear energy transfer, where the created ionisations are close to each other. The former case is applicable to electrons, while the latter is relevant for $\alpha$-particles and heavy ions. The solution to Eq. (7) is obtained after making assumptions about a Gaussian distribution of the excited states along the particle track, and considering a time *t* long enough for the triplet relaxation in the track to be predominantly diffusion-controlled, i.e., after the triplet-triplet annihilation process is no longer the dominant one. The solution provided in the work of King and Voltz is as follows:

8 $$\begin{aligned} I_d(t) = C \frac{N_T(0)}{ \left\{ 1 + \frac{\lambda _d}{\sqrt{2}t_{tt}} \left[ 1 - \left( 1 + \frac{t}{\lambda _d} \right) ^{-\frac{1}{2}}\right] \right\} ^2 \left( 1 + \frac{t}{\lambda _d} \right) ^\frac{3}{2} }, \end{aligned}$$

where $\lambda _d$ is the delayed fluorescence decay time, $N_T(0)$ is the initial concentration of triplet states and $t_{tt}$ is the triplet–triplet annihilation relaxation time. Since $t_{tt}$ is inversely proportional to *dE*/*dx*, for particles with low stopping power it could be reasonable to assume that:

9 $$\begin{aligned} \frac{\lambda _d}{\sqrt{2}t_{tt}} \left[ 1 - \left( 1 + \frac{t}{\lambda _d} \right) ^{-\frac{1}{2}} \right] \ll 1. \end{aligned}$$

Under such assumptions the equation is reduced to:

10 $$\begin{aligned} I_d(t) = \frac{M}{\left( 1+ t/\lambda _d \right) ^{\frac{3}{2}}}\,\,\,with\,\,\,M = \frac{k_fk_{tt}\tau _S N_T(0)}{2\chi _{tt}t_{tt}}. \end{aligned}$$

It should be stressed, however, that this simplification should be reasonable only for *t* that is long enough so that effects of the initial conditions are negligible. This excludes the finite rise time of the delayed scintillation light.

**Approximation of the time interval distribution to obtain the prompt fluorescence decay time**

We also assume that the time response of the PMTs is described by a Gaussian function. From the methods presented in^15^, the convolution of an exponential distribution of the type $f(x) = \tau e^{-\tau x}$ with a Gaussian distribution results in an exponentially modified Gaussian distribution with the following form:

11 $$\begin{aligned} EMG(t; \tau , \mu , \sigma ) = \frac{\tau }{2} e^{\frac{\tau }{2} \left( 2\mu + \tau \sigma ^2 - 2t\right) } \text {erfc}\left( \frac{\mu + \tau \sigma ^2 - t}{\sqrt{2}\sigma } \right) , \end{aligned}$$

where the parameters $\mu$ and $\sigma$ are the Gaussian centroid and standard deviation, erfc denotes the complementary error function $\text {erfc}(x) = 1 - \text {erf}(x)$.

These equations are used in a Python program employing the SciPy library^27^ to extract the fast component using only the first ten nanoseconds of the time distribution spectrum. The data obtained is then used as input for a Monte-Carlo code to extract the parameters of the slow component.

**Monte Carlo simulation of time distributions and validation**

This Monte-Carlo code was specifically developed using the Rust programming language^28^ to simulate the time distribution of scintillation events using input data from a scintillator. In this work, it is used to adjust the delayed component duration parameter corresponding to the measured results. The code takes several parameters as input, including those related to the scintillator and PMTs, such as:

- The S (ph.e$^{-}\cdot$keV$^{-1}$) for the prompt and delayed fluorescence,
- The decay constant (ns) of prompt and delayed fluorescence,
- The standard deviation of the Gaussian jitters,
- The relative efficiency of the PMT that we measured and calculated in our device with the method we previously developed^29^,
- Density of the scintillator (g$\cdot$cm$^{-3}$),
- kB value in (cm$\cdot$MeV$^{-1}$),
- Z/A value of the scintillator,
- The spectrum or energy of ionising radiation interacting in the scintillator.

As output, the code provides us with the distribution of events between two photomultipliers, allowing us to model the behavior of a chosen radiation and scintillator under our experimental conditions.

**Figure 22:**
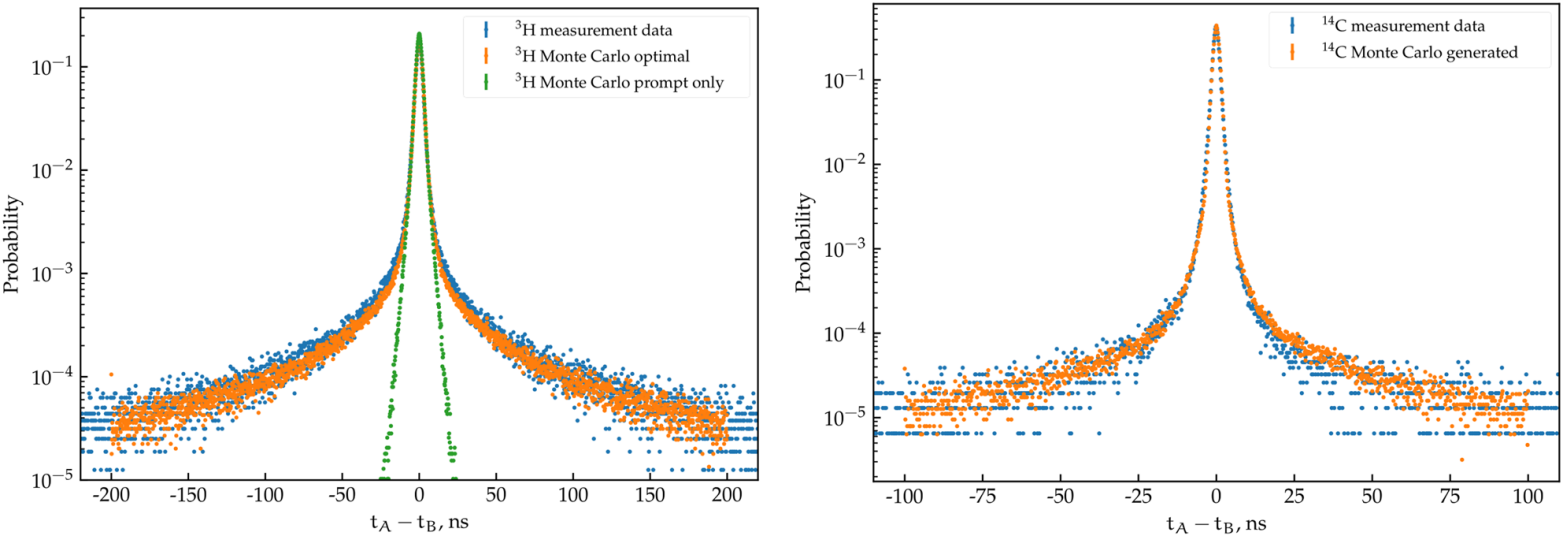
Comparison between the Monte Carlo simulation and a real measurement of $^3$H (left) and $^{14}$C (right).

To validate the code and compare it with real measurements, a study on two liquid scintillator sources was performed. The sources consisted of $^3$H and $^{14}$C in a toluene + PPO cocktail. The measurements were conducted using a TDCR LS detector connected to a CAEN DT5751 digitizer. The list-mode files were analyzed with dedicated software to obtain the distribution of the time differences between PMTs A and B, as presented in blue in Fig. 22.

The Monte Carlo code was used to generate simulated scintillation events with various input parameters. The Monte Carlo-simulated data and actual measurement data were plotted on the same graph, and simulation parameters were adjusted iteratively in a manual fashion until a satisfactory agreement was achieved.

The optimal parameters of the code that produced the best agreement between the two sets of data are as follows:

- For the prompt fluorescence: S equal to 1.0 ph.e^-^/keV, prompt decay constant of 2.5 ns, and standard deviation of the Gaussian jitter of 1.2 ns.
- For the delayed fluorescence: S equal to 0.25 ph.e^-^/keV and delayed decay constant of 10 ns.

An additional set of data was generated using the same parameters, except that the S for delayed fluorescence was set to zero, effectively removing its contribution.

The same comparison was conducted for the $^{14}$C source. The Monte Carlo parameters were optimized manually until the following was achieved: S equal to 0.7 ph.e^-^·keV^−1^ for the prompt fluorescence, prompt decay constant of 3.0 ns, S equal to 0.25 ph.e^-^·keV^−1^ for the delayed component, delayed decay constant of 10 ns, and a standard deviation of the Gaussian jitter of 0.8 ns. The only difference with the $^3$H source is a slightly lower Gaussian time jitter, a shorter prompt S, and a longer prompt decay constant. The optimal parameters found for both $^3$H and $^{14}$C are reasonable and consistent with practical observations.

As running the Monte Carlo code is computationally expensive, for now it is not possible to use an optimization method to fit the experimental curves with the MC output. Moreover, the fitting is made significantly harder due to the inherently noisy output of a Monte Carlo calculation. Therefore, this comparison is meant to be qualitative, showing that the assumptions made in the code lead, at least qualitatively, to the same results as in the experimental data. Due to the manual nature of the fitting procedure we are unable to extract uncertainties on the provided parameters, and for those, the analytical approach described in Sect. "Timing properties of liquid scintillation cocktails" should be used instead.

### Organic scintillator composition and preparation

**Liquid Scintillator**

The liquid scintillator prepared in CEA laboratory was based on toluene with dissolved 2,5-diphenyloxazole (PPO, 3 wt%) as primary fluorophore and dissolved 1,4-bis(5-phenyloxazol-2-yl) benzene (POPOP, 0.03 wt%) as wavelength shifter. Argon was bubbled through the preparation to remove any presence of quenching gas such as oxygen and then sealed in a PE-PTFE vial.

All the scintillation cocktails were used to prepare four 10 mL samples, one per each cocktail, in polytetrafluoroethylene (PTFE) coated polyethylene (PE) vials. These PE-PTFE vials, which correspond to a standard in the scintillation field, ensure a good light diffusion, and at the same time, the plastic has a lower probability to interact with $\gamma$-rays coming from the radioactive source compared to vials made of glass.

**Plastics Scintillator** were also prepared in chemistry laboratory at CEA with the following composition based on laboratory preparation procedure^30^.

- The scintillator A is a Polystyrene (PS) matrix, a first dye at 20 wt% concentration biphenyl to which is added a secondary first dye p-terphenyl (p-T) and a secondary dye POPOP. The p-T was used here to increase the pulse shape discrimination properties of the platic scintillator.
- The scintillator B was designed for $\beta$ and $\gamma$ discrimination and is composed of 81.4 wt% of PS, 17 wt% Biphenyl 1.5 wt% p-T and 0.1 wt% of POPOP.
- The scintillator C was designed to have a high scintillation yield with 97 wt% of PS, 3 wt% of PPO and 0.03 wt% of POPOP.

Each of these samples was machined to obtain a cylinder of the same size as the liquid scintillation samples, i.e. a 20 mL vial filled to 10 mL. The outer surface of the cylinder was naturally scattering due to the machining process.

## Data Availability

The datasets used and/or analysed during the current study available from the corresponding author on reasonable request.
